# Do **β**-Defensins and Other Antimicrobial Peptides Play a Role in Neuroimmune Function and Neurodegeneration?

**DOI:** 10.1100/2012/905785

**Published:** 2012-04-19

**Authors:** Wesley M. Williams, Rudy J. Castellani, Aaron Weinberg, George Perry, Mark A. Smith

**Affiliations:** ^1^Department of Biological Sciences, Case Western Reserve University, Cleveland, OH 44106, USA; ^2^Department of Pathology, University of Maryland, Baltimore, MD 20742, USA; ^3^UTSA Neurosciences Institute and Department of Biology, University of Texas at San Antonio, San Antonio, TX 78249, USA; ^4^Department of Pathology, Case Western Reserve University, Cleveland, OH 44106, USA

## Abstract

It is widely accepted that the brain responds to mechanical trauma and development of most neurodegenerative diseases with an inflammatory sequelae that was once thought exclusive to systemic immunity. Mostly cationic peptides, such as the **β**-defensins, originally assigned an antimicrobial function are now recognized as mediators of both innate and adaptive immunity. Herein supporting evidence is presented for the hypothesis that neuropathological changes associated with chronic disease conditions of the CNS involve abnormal expression and regulatory function of specific antimicrobial peptides. It is also proposed that these alterations exacerbate proinflammatory conditions within the brain that ultimately potentiate the neurodegenerative process.

## 1. Introduction

Chronic activation and impaired regulation of the innate immune response within the brain has been proposed by Fernández et al. [1] as a primary, unifying cause of Alzheimer's disease (AD) [2]. Antimicrobial peptides (AMPs) are important components of the innate immune system and also have been shown to function as regulators of the adaptive immune system [3, 4]. Williams [5] proposed that these agents may underlie neuropathologies within the human brain associated with neurodegenerative disease, aging, diabetes mellitus, and traumatic brain injury (TBI) [6]. The most extensively studied AMPs are the human *β*-defensins (hBDs), and the cathelicidin LL37/hCAP18, the principal candidate immunoregulators in extracerebral tissues [7, 8]. Herein, we present the hypothesis, based primarily on findings from extracerebral tissues, that AMPs also play a functional role in the human brain [9, 10]. Although speculative, we hope that this postulate will contribute to expanding future research into AMP function within the brain.

 A proinflammatory state within the brain is a known consequence of TBI [11], neurodegenerative diseases including Alzheimer's disease (AD) [12], senescence [13], and diabetes mellitus [14]. Despite intense research, neuroinflammatory-associated mechanisms that may underlie neuronal injury remain poorly understood. The human *β*-defensin peptides hBD-1 and hBD-2 have been isolated, and two genes encoding homologous *β*-defensin-1 and -2 (rBD-1 and rBD-2) have been identified in the rat [15]. The presence of LL37 in serum and cerebrospinal fluid extracted from patients with bacterial meningitis has also been reported. This peptide has been localized in astrocytes and microglial cells in a pneumococcal meningitis rat model [16]. *β*-defensins have been hypothesized to play a role in the etiology of neurodegeneration with a focus on traumatic brain injury, a risk factor for AD [17]. Moderate-to-severe TBI induces neuropathological responses that are similar to changes described in the AD brain, including development of chronic inflammation and hyperglycemia [18–22].

## 2. Hypothesized Role of **β**-Defensin Antimicrobial Peptides in Proinflammatory Mechanisms of Neurodegeneration

**Figure 1:**
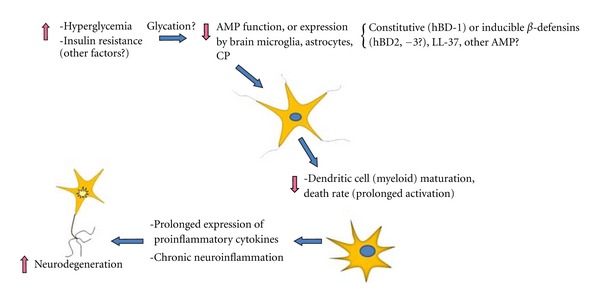
It is hypothesized that reduction or abnormal elevation of AMP expression by brain microglia, astrocytes, or choroid plexus epithelium (CP) may contribute to loss of AMP-induced regulation of dendritic cell maturation and activity. Prolonged and elevated dendritic cell activity could in turn contribute to chronic release of proinflammatory cytokines (IL-6, IL-8, etc.) that ultimately promote neuronal cell injury and death. Hyperglycemia-induced glycation of specific AMPs may inhibit antimicrobial and immunomodulatory function.

The *β*-defensins-1, -2, and -3 are recognized as principal regulators of immune response and inflammation [23–25]. Here, we propose that central to impairment of the innate and adaptive immune response, and thus prolongation of inflammation within the brain, is a dysregulation of specific AMP function, including constitutive and inducible *β*-defensin peptides (Figure 1). Attenuation in function may be attributed to an *a priori* development of an insidious condition, such as hyperglycemia and/or increased insulin resistance, a component of many neuropathological conditions including AD [26], Huntington's disease [27], aging of the brain [28], diabetes mellitus [29, 30], and TBI [31]. Reduced expression of hBD-2 and -3 mRNA occurs *in vitro* subsequent to exposure of human primary epithelial cells to high glucose (30 mM) and/or low insulin (<5 *μ*g/mL) (Williams, unpublished). These findings are consistent with the hypothesis that dysregulated expression of constitutively expressed AMPs like hBD-1 [32], specific inducible hBDs, including hBD-2 and-3, and perhaps other AMPs such as LL-37 [33], all potential modulators of inflammation, may occur in the traumatized brain and in brain tissue exhibiting chronic inflammation-associated neurodegeneration, as is observed in AD. Conversely, abnormally high levels of AMPs could also contribute to elevated and prolonged inflammation within susceptible brain regions. Chronic hyperglycemia could potentially contribute to glycation (see Figure 1) of specific amino acid residues on AMPs with formation of advanced glycation end-products (AGEs), conformational change, and ultimately inhibition of or prolongation of AMP function [34]. The intensity of a proinflammatory process subsequent to altered AMP expression level and/or function most likely would reflect the combined effects of multiple factors including the level of inflammatory cell activation, inflammatory cell density and rate of cell turnover, and effectiveness of compensatory anti-inflammatory modulators such as microglial intervention and upregulation of the anti-inflammatory cytokine IL-10 [35]. Thus, the induction of inflammation within susceptible brain regions of chronic diabetics presenting with poor glycemic control (hyperglycemic) and in chronic neurodegenerative diseases may exhibit chronic, but low-grade inflammation [36, 37] that may not be substantially greater than that observed in more acute models of inflammation.

## 3. Mild TBI and More Severe Head Trauma Induce Cellular Injury and an Inflammatory Response in the Brain

Neuropathological alterations in neurons are sustained not only in moderate and severe TBI, but also in mild TBI, as indicated by metabolic depression indicative of neuronal injury [38], and other metabolic abnormalities [39–41]. Immune response and inflammation are considered primary to the progression of closed head injury, yet underlying mechanisms are not well understood. It is now recognized that immune function and immunomodulatory capacity are not limited to those cells classically defined as “immune” cells; so-called “nonimmune” cells also are critical to the immune response. In the brain, astrocytes, epithelium of the choroid plexus, and meningothelial cells express and likely secrete immunomodulatory antimicrobial peptides, including hBDs-1 and -2, that could influence inflammation within the brain [9, 42]. Astrocytes, for instance, are capable of responding to bacterial pathogens, including *Staphylococcus aureus* [43]. The responsiveness of these cells may be region specific, as is the case with regional responsiveness to lipopolysaccharide (LPS), and basal profile of induced cytokine expression [44–46]. Thus, astrocytes, although not immune cells according to classical definition, may significantly affect central nervous system (CNS) immune response in the pathological state, not only by variable expression and release of proinflammatory cytokines such as IL-1*β*, but also by expression of immunomodulating AMPs [47–50].

 Because of the dynamic nature of the response and the complexity of functional interrelationships between expressed immunomodulators, the role that the innate immune response and inflammation play in neuropathology remains ill-defined. Some components of the immune response, including cell-mediated immunity concurrent with reduced levels of key immunoregulatory cytokines, may be suppressed leading to exacerbated inflammation following severe head injury [51], and perhaps in neurodegenerative diseases in general. Still unclear is how the immune response contributes to secondary brain injury following TBI, and to what extent components of the response mediate not only neuronal, but also astrocytic damage. Although specific cytokines may be expressed at reduced levels following TBI, the development of secondary and tertiary changes in brain cell function can be accompanied by increased expression of proinflammatory cytokines. For example, upregulation of interleukin-1 (IL-1*α* and *β*) and tumor necrosis factor (TNF)-*α*, both components of the chronic inflammatory response, occurs with onset of depression [52, 53] and headache [54]; both are frequently described as late clinical sequelae of TBI.

 TBI induces a cell-mediated immune response within the brain and systemic circulation [55]. Under physiological conditions, the blood-brain barrier (BBB) limits movement of immunocompetent cells into the brain thus regulating the immunological response [56]. TBI-induced disruption of the BBB can lead to unregulated translocation of leukocytes, including monocytes, into the affected brain region with subsequent activation of microglia and astrocytes [57], concomitant with elevated expression of proinflammatory cytokines [58, 59]. However, both astrocytes and microglia also can limit or actively suppress inflammation by the secretion of anti-inflammatory factors such as IL-10, thereby affecting the immunosuppressive response with neuroprotective consequences [48, 60, 61].

## 4. TBI-Induced Hyperglycemia Exacerbates Cellular Damage and Inflammation in the CNS

Acute hyperglycemia is a component of the normal stress response and can exacerbate brain injury by promoting oxidative stress [62] with potentiation of the inflammatory response [63–65]. Overall, neurological outcome worsens after trauma or stroke when glucose levels are even acutely elevated [66, 67]. Acute hyperglycemia with a serum glucose level of ≥170 mg/dL is associated with poor outcome in head trauma patients who present with a Glasgow Coma Scale (GCS) of 8 or lower [68].

 Elevated serum glucose can occur in mild concussive head trauma in humans [69] and in mild, moderate, and severe head trauma in rats [70]. Moreover, extracellular glucose is significantly increased in the hippocampus and cerebral cortex ipsilateral to the site of fluid-percussion injury in the rat [71].

 Acute onset of hyperglycemia (within 1 hour of injury) contributes to cerebral neuropathology [72]. The presence of even mild-to-moderate serum glucose elevation (≥150 mg/dL) is detrimental to vulnerable brain regions [73], such as the cerebral cortex and hippocampus [74, 75]. These regions mediate self-regulation, emotion, social functioning, and working memory, all cognitive functions adversely affected by TBI [76]. Neurons and astrocytes are both susceptible to hyperglycemia-induced injury [77, 78].

## 5. **β**-Defensins Are Mediators of the Innate Immune Response and Modulate Inflammation

The defensin family of immunomodulatory peptides is an evolutionarily conserved group of cationic peptides that contribute to innate host defense. Constitutively expressed hBD-1 and inducible hBD-2 and -3 are expressed by epithelium and epithelial-derived cells [79–83]. All three hBDs can induce cellular expression of cytokines and chemokines; however, hBD-2 and -3 are particularly important mediators of cytokine release, including release of IL-6, MIP-3*α*, MCP-1, and RANTES through activation of the G protein and phospholipase C-dependent pathways [84]. These hBDs and LL-37 also promote epithelial cell migration and proliferation, angiogenesis, chemotaxis, and wound repair [84, 85]. Recruitment of specific cells such as immature dendritic cells and memory T cells involves the binding to or modulation of the chemokine receptor CCR6 by hBDs-1, -2, or -3 [86–88]. Some hBDs can also interact with other chemokine receptors, including CCR2 [89] and the G protein-coupled receptor CXCR4 [4]. These same receptors are also expressed by the immunocompetent astrocytes and microglia of the human brain [90, 91]. The hBDs-1 and -2 can be expressed by astrocytes and microglia of both human [9] and rat brains [92, 93]. The capacity of resident astrocytes and microglia to express hBD-1 is consistent with our hypothesis that hBDs, through their ability to activate immune cells and modulate cytokine release [84, 94], may function to regulate inflammation within the brain. Tiszlavicz and colleagues have recently demonstrated the expression of hBD-2 mRNA and synthesis of hBD-2 protein in human brain capillary endothelium following *in vitro* exposure to *Chlamydophila pneumoniae *[95]. Therefore, the immunoregulatory function of the *β*-defensins may be established by the microenvironment and stimulus experienced by the cell expressing the peptide. Several lines of evidence support both a pro- and anti-inflammatory role for some antimicrobial peptides. These peptides, including hBD-2, and cathelicidin LL-37 may promote the release of both proinflammatory (IL-6, IL-18) and anti-inflammatory (IL-10) cytokines and chemokines from epithelial cells as shown by several *in vitro* studies [84, 96]. Donnarumma et al. [97] also have shown in the human lung cell line A549 that hBD-2 alone or in combination with moxifloxacin can reduce the expression of IL-1*β*. Overexpression of rat *β*-defensin-2 (rBD-2) in the lungs of Sprague Dawley rats challenged with *P. aeruginosa* results in reduction of pulmonary inflammation [98]. If the *in vitro* findings presented above accurately reflect antimicrobial function *in vivo*, then a dysregulation of cerebral expression of specific antimicrobial peptides may favor either exacerbation or amelioration of the inflammatory response within the brain depending upon which extracellular conditions predominate. With respect to the *β*-defensins, we propose that hBD-1 and -2 can contribute to either a pro- or anti-inflammatory modulation of the neuroimmune response with dependence upon the temporal cellular expression of respective cytokines and chemokines. The normal expression profile, and therefore function of specific hBDs, may be compromised when the brain is subjected to mechanical trauma or disease. For example, the level of constitutively expressed hBD-1 is selectively upregulated in differentiating, but not proliferating, keratinocytes [99].

 Brain trauma is followed by onset of the acute phase stress response and development of acute hypermetabolism within the brain [100]. With moderate-to-severe TBI, increasing insulin resistance leads to a delayed but chronic hypometabolic state that promotes extracellular hyperglycemia [31]. An *in vitro* study of human embryonic kidney (HEK) cells by Barnea et al. [101] has shown that increasing intracellular glucose availability increases the expression of hBD-1. However, in rodent models of diabetes mellitus type II, hBD level, including that of hBD-1, was low compared to nondiabetic controls. This apparent discrepancy could be corrected by treatment of animals with insulin [102]. Both diabetes and TBI lead to increased insulin resistance and extracellular hyperglycemia. Insulin is required by astrocytes for proper metabolic function [103, 104]. Therefore, brain regions susceptible to the development of hyperglycemia and insulin resistance might affect the inflammatory state of the involved tissues by modulating expression of AMDs, including *β*-defensin peptides.

## 6. **β**-Defensins Are Expressed in Astrocytes, Microglia, and in Epithelium of the Choroid Plexus

TBI leads to activation of astrocytes and microglia as part of the innate immune response [105]. Astrocytes are strategically sited to provide early defense and modulatory control of cellular events following cerebral insult. The gene chip analysis by Falsig et al. [106] is of particular interest as it shows that a number of genes expressed in murine astrocytes are linked to antiviral/antimicrobial defense and are coordinately regulated. The analysis did not assess either *β*-defensins or the cathelicidin LL37. Nevertheless, these findings, together with the reported induction of hBD-2 in human astrocytes by cytokines [9], are consistent with the hypothesis that hBD-2 might function as an initial line of defense within the CNS either as an antimicrobial, immunomodulator, or both. Astrocytes, microglia, and the choroid plexus constitutively express hBD-1 [42], again suggesting that hBDs could play a role in the innate immune response of the traumatized brain.

 Currently, there are no reported studies on hBD expression or function within the human brain. Hyperglycemia [70], concomitant with increased insulin resistance, is an early feature of TBI in the acute phase response to trauma [107]. It is noteworthy that the level of trauma needs not be severe to elicit a marked elevation in cortical glucose within 24 hours [108], and even mild hyperglycemia can adversely affect brain function [109]. Although hyperglycemia remains a primary focus of many studies, insulin resistance might also contribute significantly to cellular dysfunction following trauma. Insulin receptors are widely distributed within the brain with their highest densities in cerebral cortex, hypothalamus, and hippocampus [110]. Because glucose incorporation into astrocytes is insulin sensitive [111], astrocytes could be particularly prone to trauma-induced dysfunction although some signaling pathways associated with hippocampal neurons also are insulin dependent [112, 113]. Acute hyperglycemia and insulin resistance can result in alteration of the immune response in favor of a proinflammatory condition leading to more extensive or prolonged inflammation [64].

## 7. **β**-Defensins May Be Neuroprotective through Their Ability to Impede Cellular Apoptosis, Increase Cellular Proliferation, and Promote CNS Wound Healing

Apoptosis contributes to delayed neuronal cell death in TBI [114]. Mild TBI in the lateral fluid percussion rat model has induced apoptotic TUNEL(+) astrocytes and neurons in both grey matter of the cortex and in underlying white matter [115, 116]. There is evidence that *β*-defensins *in vitro* can attenuate the onset of proapoptotic mechanisms in human neutrophils where hBD-3 can reduce apoptosis via the chemokine receptor CCR6 [117].

 TBI may increase apoptosis and also enhance reactive astrocytosis [118]. Of importance is the observation that ablation of proliferating reactive astrocytes with the antiviral agent ganciclovir significantly increases neuronal degeneration and the inflammatory state [119]. Studies *in vivo* indicate that chronic activation of the innate immune response can induce neuronal injury [120–122], perhaps through activation of the inflammasome complex [123]. Thus, modulation of this response, perhaps by specific hBDs and/or other immunomodulatory antimicrobial peptides, could be critical to the maintenance of neuronal health. Moreover, the potential contribution of *β*-defensins to reparation of inflammation-induced cellular injury in the CNS is illustrated by the ability of hBD-2 to stimulate proangiogenic activity, as demonstrated in human umbilical vein endothelial cells despite absence of growth factors such as VEGF [23]. These observations* in vitro*, although limited to conditions and to cells not directly related to those within the CNS, are nevertheless consistent with a proposed function for hBD-2 within the brain that is supported by the inducible expression of this peptide in human astrocytes stimulated by the proinflammatory cytokines IL-1*β* and TNF-*α* [9].

## 8. Dendritic Cells Are Critical Modulators of the Innate and Adaptive Immune Response

Dendritic cells (DCs) are antigen-presenting cells (APCs) crucial to pathogen recognition and regulation of the inflammatory response. DCs are critical to the induction and regulation of adaptive immunity through their release of cytokines, and subsequent activation of lymphocytes, polarization of T-helper type 1 (Th1) CD4+ cells, development of cytolytic T cells, and expansion of the antibody response to antigen [124, 125]. Upregulation of toll-like receptors (TLRs) by pathogen-associated molecular patterns (PAMPs) can induce maturation of DC to an inflammatory phenotype that upregulates the adaptive immune response [126]. Under physiological conditions, dendritic cells are restricted to the meninges and choroid plexus of the brain and are not normally present within the brain parenchyma [127]. However, rapid accumulation of DCs proximal to the site of brain inflammation occurs with neurodegeneration [128], including AD [129], autoimmune disease, bacterial and viral infection, and brain trauma [128, 130, 131]. Recent studies suggest that DCs exhibit a regulatory function with regard to the neuroinflammatory process [132] and can express both pro- and anti-inflammatory cytokines [133]. In this regard, maintenance of an active DC population may contribute to prolongation of the inflammatory process [134–136] with progressive neurodegeneration.

## 9. **β**-Defensin Peptides May Modulate Both Innate and Adaptive Immune Responses within the Brain through Chemotaxis and Promotion of Dendritic Cell Maturation

The antimicrobial function of AMPs might actually be secondary to their immunomodulatory capabilities [137]. One mechanism by which the initial innate response can activate an adaptive response is through chemoattraction, maturation, and activation of dendritic cells [138]. At least some AMPs, including *β*-defensins-1, -2, and -3, and LL-37 can chemoattract immature dendritic cells and induce their maturation [139–142]. Yang et al. [86] have shown that human *β*-defensin-2 is chemotactic for immature DCs through binding to the DC-expressed CCR6 receptor.

## 10. **β**-Defensin Peptides May Limit Inflammation through Anti-Inflammatory Pathways and by Induction of Dendritic Cell Death

The ability of specific *β*-defensin peptides to regulate inflammation may rely not only on promotion of adaptive immunity and inflammation, but also on curtailing the inflammatory process itself. Chronic inflammation is counterproductive, often contributing to excessive cell death, and compromising tissue repair. Hao et al. [143] have demonstrated improved wound healing in cultured bone-marrow-derived mesenchymal cells (BMSCs) expressing both human platelet-derived growth factor-A (hPDGF-A) and hBD-2. Pingel et al. [144] also show that hBD-3 can attenuate the proinflammatory cytokine response to microbial antigens by myeloid dendritic cells exposed to recombinant hemagglutinin B (rHagB), one of five hemagglutinins that promote binding of *Porphyromonas gingivalis* (*P. gingivalis*) to host cells, including myeloid DCs. The mechanism by which hBD-3 attenuates the IL-6, IL-10, and TNF-*α* response in human myeloid dendritic cells was not determined. However, Pingel et al. demonstrated strong binding between the highly cationic hBD-3 and immobilized rHagB. Furthermore, significantly lower levels of ERK 1/2, but not JNK 1/2 or p38, were observed, suggesting a partial involvement of the MAPK pathways, important to control the inflammatory response to *P. gingivalis*. The authors propose that binding of hBD-3 to rHagB may be an initial step leading to suppression of select proinflammatory cytokine pathways in human myeloid dendritic cells. Recently, Semple et al. [145] demonstrated that hBD-3 attenuates transcription of proinflammatory genes in TLR4-stimulated macrophages. Whether these results are relevant to defensin function within the brain is unknown. Such a functional versatility on the part of specific defensins would be highly advantageous to controlled modulation of the immune response within the CNS.

*β*-defensins might also modulate the duration of inflammation by controlling the viability of activated macrophages and the same dendritic cells that they chemoattract and induce to maturity. In an interesting study of TLR-4-dependent maturation of immature DCs by murine *β*-defensin-2 (mBD-2), Biragyn et al. demonstrated that following activation of DCs, mBD-2, not orthologous to any known hBDs, induces DC death via an NF*κ*B-dependent induction of  TNF-*α* and TNF receptor 2 (TNFR2) expression on the APC surface [146]. Cytotoxicity was shown to be due to a defensin-induced signaling cascade that requires TNFR2 and not mBD-2 directly. Additional experiments by this group clearly showed that these observations were not restricted to murine *β*-defensin, since hBD-3 also was capable of inducing, first, DC maturation and then death several days later.

 The studies noted above strongly suggest that at least some hBDs, under certain conditions, possess an anti-inflammatory or inflammation-limiting capability. Conditions under which these defensin-related anti-inflammatory mechanisms are activated *in vivo* and whether they function within the CNS remain to be determined. The pro- or anti-inflammatory nature of hBDs may depend largely on temporal regulation of cellular events, including number and type of inflammatory cells (microglial activation state) and molecular expression and inactivation of pro- or anti-inflammatory cytokines and chemokines by immunocompetent cells within the cellular microenvironment.

## 11. Summary and Concluding Remarks

A number of neurodegenerative diseases, including AD, the aging process, TBI, and type II diabetes mellitus, exhibit similar pathogenic cascades. Hyperglycemia and elevated insulin resistance are common sequalae, among others, that may underlie cellular dysfunction within the brain involving altered expression of immunomodulatory peptides, such as the *β*-defensins. We propose, based on studies that support a central role for *β*-defensins as modulators of immune response and inflammation in extracerebral tissues, that these peptides, and perhaps other AMPs endogenous to the human brain, contribute to regulation of the immune response and inflammation within the CNS. This would likely occur in part through the ability of specific antimicrobial peptides, such as *β*-defensins-1, -2, and -3, to regulate dendritic cell function and viability. We also propose that neuropathological development of hyperglycemia, and/or insulin resistance, attenuates *β*-defensin function through reduced cellular expression of the peptide and compromised ability to modulate the neuroimmune response, perhaps through uncontrolled dendritic cell activation. However, AMP function is unlikely to be influenced by a single mechanism. Given the complex etiology of neurodegenerative diseases, it is likely that peptide activity is a function of multiple factors inherent within specific regions of the brain. The net effect on AMP function may be reflected in increased pro- or anti-inflammatory activity by the AMP peptide(s) that results in an intensified or prolonged inflammatory process. The recent study by Soscia et al. [147], for example, suggests that the oligomerized form of *β*-amyloid 1–42 (A*β*), an anionic peptide associated with AD neuropathology, may function as a proinflammatory antimicrobial peptide in regions of the brain exhibiting elevated levels of the peptide. Oligomerization of monomeric hBD-2 also has been demonstrated and is likely to occur with other hBDs and AMPs [148]. Many AMPs exhibit structural characteristics, including a *β*-sheet conformation similar to A*β*(1–42) that contribute to oligomerization. Yet while some AMPs like frog skin dermaseptin S9 form amyloid-like fibrils [149], others such as hBD-2 and -3 do not [150]. Interestingly, as noted by these studies, amyloidogenic properties may adversely affect normal peptide function and favor a proinflammatory state, while nonamyloidogenic oligomerization appears to promote antimicrobial function, including that of the *β*-defensins without promoting inflammation. Increased or decreased *β*-defensin expression and function could contribute significantly to chronic inflammation and ultimately to the neurodegenerative process itself. Clearly, regulation of the innate immune response in tandem with upregulation of cellular repair processes within the CNS is complex and highly integrated with considerable redundancy. Nevertheless, the proposal that AMPs function within the brain is supported by the cytokine-induced expression of AMPs by human astrocytes and microglial and the functional activity of AMPs in multiple organ systems, as described above. Whether specific AMPs play a central role in the onset or promotion of the neuroinflammatory process and neurodegeneration is currently unknown, emphasizing the importance of further investigation into the regulatory mechanisms that control innate and adaptive immunity within the CNS.
